# The role of wild canids in the epidemiology of *Neospora caninum*: a systematic review

**DOI:** 10.1016/j.ijppaw.2026.101319

**Published:** 2026-11-04

**Authors:** Agustina Soto-Cabrera, Nathalia P. Scioscia, Dadin P. Moore, Gastón Moré

**Affiliations:** a Instituto de Innovación para la Producción Agropecuaria y el Desarrollo Sostenible (IPADS Balcarce), Instituto Nacional de Tecnología Agropecuaria Estación Experimental Agropecuaria Balcarce (CONICET-INTA), Ruta 226, km 73.5, Buenos Aires, Balcarce, 7620, Argentina; b Instituto de Investigaciones en Producción, Sanidad y Ambiente (IIPROSAM), Facultad de Ciencias Exactas y Naturales, UNMdP, Funes, 3350, Mar del Plata, Argentina; c Consejo Nacional de Investigaciones Científicas y Técnicas (CONICET), Argentina; d Facultad de Ciencias Agrarias, Universidad Nacional de Mar del Plata, Ruta 226, km 73.5, Buenos Aires, Balcarce, 7620, Argentina; e Institute of Parasitology, Department of Infectious Diseases and Pathobiology, Vetsuisse Faculty, University of Bern, Länggassstrasse 122, 3012, Bern, Switzerland

**Keywords:** Definitive host, Intermediate host, Oocysts, Serology, PCR

## Abstract

*Neospora caninum* is a globally distributed apicomplexan parasite for which dogs (*Canis lupus familiaris*) and some wild *Canis* species serve as definitive hosts (DH), and it has a complex life cycle involving a wide range of animals, particularly ruminants, as intermediate hosts (IH). Despite its epidemiological relevance at the wildlife–livestock interface, a comprehensive overview of the role of wild canids in the parasite's life cycle is lacking. Following the PRISMA 2020 guidelines, this systematic review aimed to: (i) assess the evidence supporting the dual role (DH and IH) of wild canids in the *N. caninum* cycle, (ii) evaluate the methodologies and diagnostic criteria used to confirm DH status, and (iii) analyse global patterns of exposure, tissue infection, and transmission. From 732 records screened, 74 studies met inclusion criteria. Twenty articles investigated wild canids as DH, but oocyst shedding was confirmed in only three: *C. latrans*, *C. lupus dingo*, and *C. lupus*. Sixty-seven studies assessed exposure in 19 species, with *Vulpes vulpes* being the most extensively monitored; seroprevalence varied widely by species and regions. Molecular and histopathological evidence confirmed tissue stages in several species, including *Lycalopex gymnocercus*, supporting their role as IH, while vertical transmission further indicates their contribution to maintaining sylvatic cycles.

Overall, wild canids contribute more substantially as IH than DH in the epidemiology of *N. caninum*. Future research should standardize diagnostic approaches and expand sampling in underrepresented regions, particularly Africa, Asia, and South America.

## Highlights

• Definitive host diagnosis requires oocyst detection and molecular confirmation.
• Only three *Canis* species are confirmed as definitive hosts.
• Wild canids contribute to the epidemiology of *N. caninum*, primarily as intermediate hosts.
• Methodological heterogeneity limits comparability across studies.
• Major knowledge gaps persist in underrepresented regions and canid species.

## Graphical abstract

**Figure:**
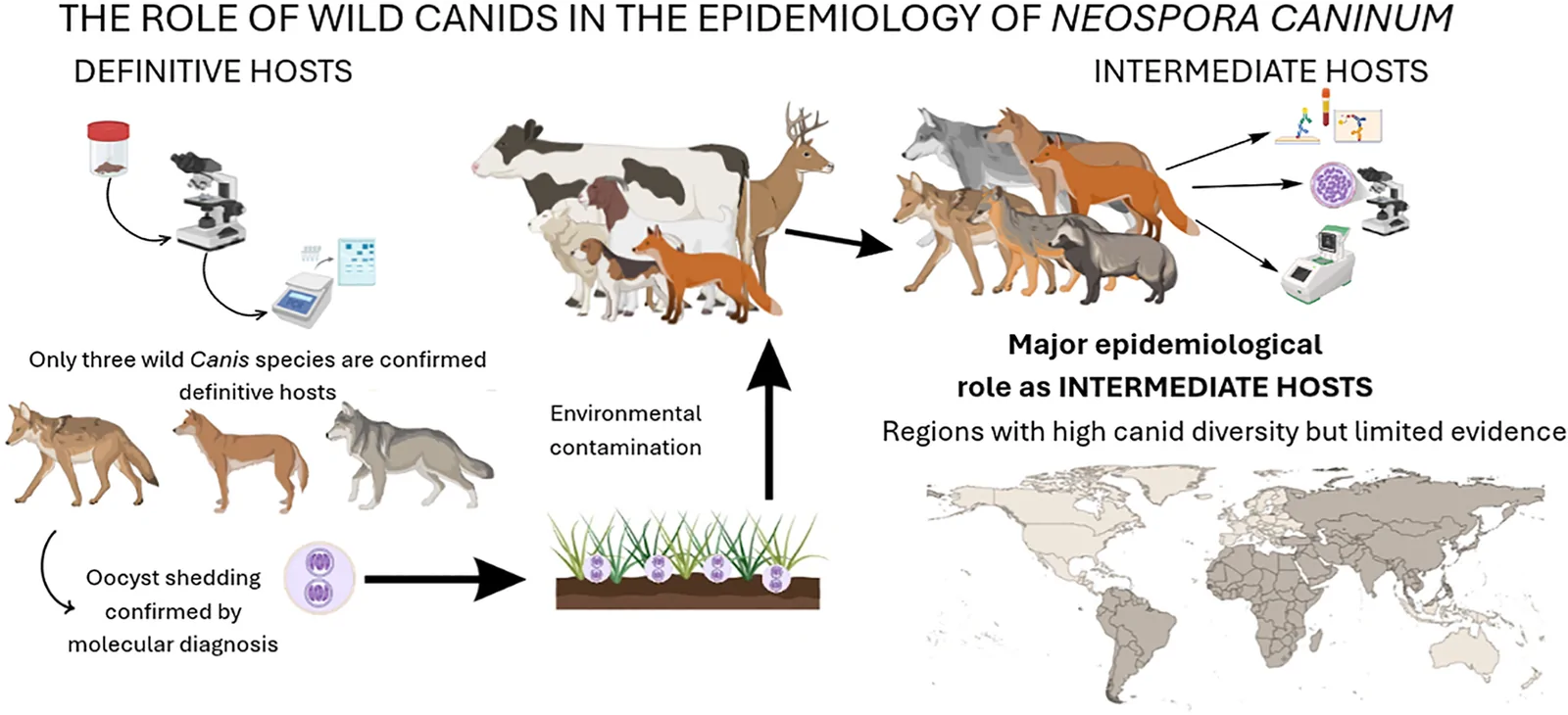

## 1 Introduction

Neosporosis is a parasitic infection caused by *Neospora caninum*, an obligate intracellular protozoan belonging to the phylum Apicomplexa and the family Sarcocystidae. This parasite affects a wide range of warm-blooded animals (Dubey et al., 2007) and is recognised worldwide as the cause of abortions in cattle and severe neurological disorders in dogs (Buxton et al., 2002; Dubey et al., 2017). It has a heteroxenous and facultative life cycle, with domestic dogs and other species of the genus *Canis* acting as definitive hosts (DH), while numerous mammals, including canids, act as intermediate hosts (IH) (Dubey et al., 2017).

Neosporosis was first described by Bjerkås et al. (1984) in puppies from Norway with lesions in the central nervous system (CNS) and cysts in muscles. Subsequently, McAllister et al. (1998) identified the domestic dog (*Canis lupus familiaris*) as DH, and the elimination of oocysts in faeces was later reconfirmed (Lindsay et al., 1999). Following this, Basso et al. (2001) isolated oocysts from one naturally infected dog in Argentina. Additionally, other wild species have been confirmed as DH, including the coyote (*C. latrans*) (Gondim et al., 2004a), the dingo (*C. lupus dingo*) (King et al., 2010), and the grey wolf (*C. lupus*) (Dubey et al., 2011).

The life cycle of *N. caninum* includes three infectious stages: sporozoites (in sporulated oocysts shed by DH), tachyzoites (rapid multiplication stage in nucleated cells) and bradyzoites (slow multiplication stage within tissue cysts, mainly in the CNS and muscles) (Dubey et al., 2017). DHs become infected mainly by ingesting tissue cysts and shed immature oocysts that sporulate in the environment (Gondim et al., 2004b; McAllister et al., 1998). In IH, transmission can be horizontal (ingestion of sporulated oocysts) or vertical (transplacental), when tachyzoites are transferred from the mother to the fetus (Dubey et al., 2017).

Oocyst ingestion represents the only confirmed postnatal transmission route in herbivores, and their persistence in the environment makes this phase critical for the epidemiology of the parasite (Dubey et al., 2017). Although wild canids could maintain infection via vertical transmission and harbour tissue stages (Schares et al., 2001; Zanet et al., 2023), their role as definitive hosts appears limited; oocyst shedding has been documented in only a few individuals and in low quantities over short periods (Gondim et al., 2004a; King et al., 2010).

In canids acting as IH, the clinical presentation has been described mainly in dogs, with neuromuscular symptoms in congenitally infected puppies: paralysis, weakness, rigidity, and muscle atrophy in the hind limbs (Bjerkås et al., 1984; Buxton et al., 2002). Systemic signs such as hepatomegaly, megaesophagus, lymphadenopathy, and enteropathy, and multifocal nodular dermatitis have also been reported (Alf et al., 2024; Basso et al., 2005; Boyd et al., 2005; Dubey et al., 2017).

The most recent reviews on neosporosis in wildlife are those by Almería (2013), Donahoe et al. (2015) and Dubey et al. (2017), which emphasise the epidemiological significance of dingoes, coyotes and wolves in the parasite cycle and provide serological data on multiple canid species. However, since these publications, significant advancements in molecular diagnostic techniques and a growing body of field data have emerged, requiring an updated analysis of the status of wild species. Moreover, there is an absence of a review that systematically outlines the methodology employed to substantiate the role of wild canids as DHs, a fundamental aspect for comprehending the dissemination of oocysts within environmental contexts.

Consequently, this study aimed to:(1) systematically review the evidence supporting the dual role (DH and IH) of wild canids in the *N. caninum* cycle worldwide; (2) critically evaluate the methodologies and diagnostic criteria used to substantiate DH status; and (3) assess global patterns of exposure, tissue infection, and transmission dynamics in these species.

## 2 Methods

This study was designed following the PRISMA 2020 statement for a Systematic Review (PRISMA, 2020). We performed a systematic and structured search on 18 November 2025 to find articles using Scopus as a bibliographic database. The search was done with the terms: [(Wild AND cani*) OR lycalopex OR canis AND (Neospora AND caninum)]. To avoid missing data, we also checked the bibliographic references of the most relevant published papers.

### 2.1 Inclusion and exclusion criteria

Study eligibility was defined based on specific inclusion and exclusion criteria. Inclusion criteria comprised: (1) original research articles with the focus on *N. caninum* in wild canid species; (2) studies providing primary data on DH roles or infection exposure; and (3) papers published in English. Conversely, exclusion criteria were applied to: (1) review articles, (2) studies exclusively involving domestic dogs (*C. lupus familiaris*), (3) research on other non-canid species, and (4) studies where the primary focus was not *N. caninum*.

### 2.2 Study selection and data extraction

Following the initial search, metadata (including author, title, year, abstract and DOI) were exported to an Excel database. The screening process consisted of a two-stage evaluation: first, a title and abstract review to identify potentially relevant studies, followed by a full-text analysis of the selected papers. In order to systematize the information, a Google Form was used to standardize the data extraction. This approach facilitated the systematic documentation of key variables, including: (1) study objective and design; (2) target canid species and its geographic origin; (3) sample size and matrix type (e.g. serum, faeces, tissue); (4) specific diagnostic methodology used to determine the DH role or exposure to infection; and (5) principal findings and conclusions. Furthermore, an additional search was conducted in PubMed using the same search terms and eligibility criteria applied in Scopus.

## 3 Results

The systematic search yielded an initial total of 732 records. Following a screening of title and abstract, 81 articles were selected for full-text eligibility assessment. Papers were included if they provided primary data on the role of wild canids as either DH or IH, while those that focusing on diagnostic tests validation or unrelated topics were excluded. An additional search in PubMed identified 296 records. After title and abstract screening, 58 records were considered relevant; however, all studies meeting the inclusion criteria had already been identified through Scopus, and no additional articles were included in the review.

The final selection comprised 74 articles. Notably, an exception was made to include one highly relevant original article published in German. The included studies were categorized into two main thematic strands: (1) research evaluating the role of wild canids as DH; (2) research assessing parasite exposure and infection in their role as IH. Because several articles contributed data to both categories, these roles are analysed independently in the following sections to systematically organize the methodologies and findings (Fig. 1).

**Fig. 1:**
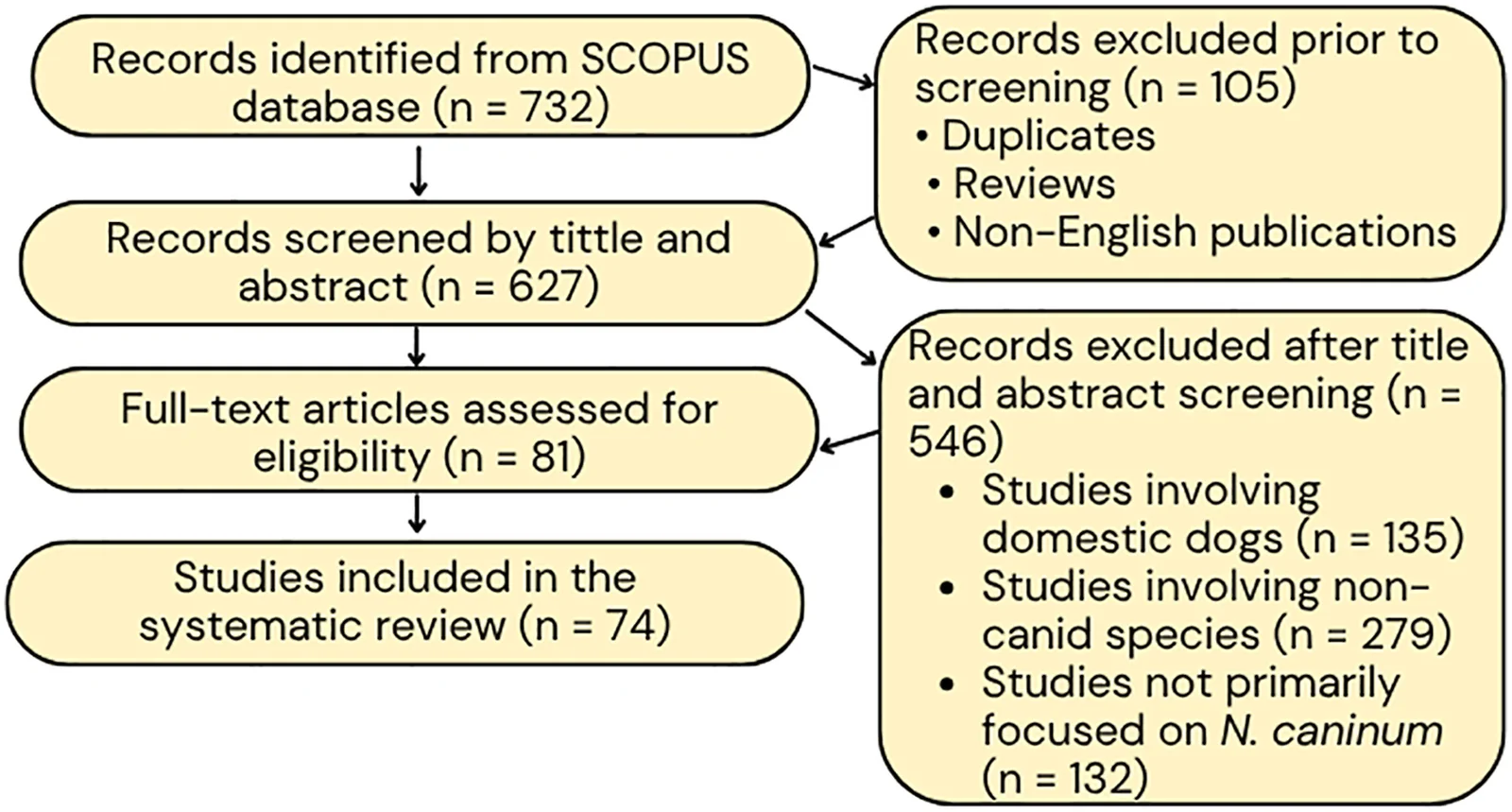
**Flow diagram of study identification, screening, eligibility assessment, and inclusion in the systematic review.** Records were identified through SCOPUS searches and screened according to predefined eligibility criteria.

### 3.1 Wild canids as definitive host

Twenty of the selected papers investigated wild canid species as DH for *N. caninum* (Almeida et al., 2019; Almería et al., 2002; Bandelj et al., 2023; Constantin et al., 2011; Davidson et al., 2022; Dubey et al., 2011; Gondim et al., 2004a, 2018; Hermosilla et al., 2017; King et al., 2010; Klein et al., 2019; Lindsay et al., 1996; Mannise et al., 2021; Mohammed et al., 2003; Moreno-Torres et al., 2018; Perrucci et al., 2023; Schares et al., 2002; Soares et al., 2009; Stuart et al., 2013; Wapenaar et al., 2006) (Table 1).

**Table 1:** Description of studies researching wild canids as definitive host of *N. caninum*.

Wild canid species	Country	Type of Study	N	Faeces samples		Coprological analysis		Molecular analysis					Observation	Author
				Conservation	Type of Diagnosis	Methods	N (+)	Purification	Methods	Target	Primers	N (+)		
*Canis aureus* (Golden Jackal)	Slovenia	NIM	39	Frozen	PCR			No	Real time	pNc5	NP1/NP2	1		Bandelj et al. (2023)
*Canis lupus dingo* (Dingo)	Australia	EIM	4	N/A	Coproparasitology PCR	Salt	1	No	Conventional	pNc5	Np6 plus/Np21 plus	1		King et al. (2010)
*Canis lupus dingo hibrids* (Hibrid dingo-dog)	Australia	NIM	19	Frozen	PCR			No	Convectional	pNc5	Np6 plus/Np21 plus	2		Davidson et al. (2022)
									Nested	ITS1 rDNA	NN1/NN2; NP1/NP2	0		
*Canis latrans* (Coyote)	Canada	NIM	100	Frozen	PCR				Real time	pNc5	NC5-550/NC5-596	10		Klein et al. (2019)
		NIM	185	Processed immediately	Coproparasitology PCR	Sucrose	2	No	Convectional	pNc5	Np6/Np21	2	95-99% identity *to N. caninum*	Wapenaar et al. (2006)
	United States	NIM	225	N/A	Coproparasitology PCR	Sucrose	N/A	No	Convectional	pNc5	Np6 plus/Np21 plus	0		Moreno-Torres et al. (2018)
		EIM	4	Processed immediately	Coproparasitology PCR	Sucrose	1	Yes	Convectional	pNc5	Np6/Np21	1	*Hammondia heydorni* (−)	Gondim et al. (2004a)
		EIM	3	Refrigerate	Coproparasitology	Sucrose	0						Bioassay (−)	Lindsay et al. (1996)
*Canis lupus* (Wolf)	Croatia	NIM	400	Alcohol	Coproparasitology	SAF	10							Hermosilla et al. (2017)
	United States	NIM	73	Processed immediately	Coproparasitology PCR	Sucrose	3	Yes	Convectional	pNc5	Np6 plus/Np21 plus	1	Bioassay and Cell culture (+)	Dubey et al. (2011)
	Italy	NIM	79	Frozen	Coproparasitology	Salt	1							Perrucci et al. (2023)
	Slovenia	NIM	42	Frozen	PCR			N/A	Real time	pNc5	NP1/NP2	3		Bandelj et al. (2023)
*Cerdocyon thous* (Crab-eating fox)	Brazil	NIM	25	Frozen	Coproparasitology PCR	Sucrose	0	No	Nested	ITS1 rDNA	NN1/NN2; NP1/NP2	13		Almeida et al. (2019)
		EIM	4	Processed immediately	Coproparasitology PCR	Sucrose	2	No	Seminested	pNc5	Np4/Np7,Np6/Np7	0	*Hammondia heydorni* (+)	Soares et al. (2009)
*Lycalopex gymnocercus* (Pampas fox)	Uruguay	NIM	14	Refrigerate	Coproparasitology PCR	Ritchie sedimentation	0	No	Real time TaqMan		Nc-UY forward, Nc-UY reverse	1	Reporter FAM. Quencher ZEN/IBFQ	Mannise et al. (2021)
*Lycalopex vetulus* (Hoary fox)	Brazil	NIM	1	Processed immediately	Coproparasitology PCR	Sucrose	1	No	Conventional	pNc5	Np6 plus/Np21 plus	0	*Hammondia* sp*.* (+)	Gondim et al. (2018)
*Vulpes vulpes* (Red fox)	Australia	NIM	50	Frozen	PCR			N/A	Convectional	pNc5	Np6 plus/Np21 plus	0		Davidson et al. (2022)
	Canada	NIM	271	Processed immediately	Coproparasitology PCR	Sucrose	2	No	Convectional	pNc5	Np6/Np21	2	95-99% identity to *N. caninum*	Wapenaar et al. (2006)
	Germany	EIM	8	Processed immediately	Coproparasitology PCR	Sucrose	7	Yes	Convectional	pNc5	Np6 plus/Np21 plus	0	*Hammondia heydorni* (+)	Schares et al. (2002)
		NIM	2191	Frozen	Coproparasitology PCR	Sucrose	65	No	Convectional	pNc5	Np6 plus/Np21 plus	0	*Hammondia heydorni* (+)	Constantin et al. (2011)
	Italy	NIM	101	Frozen	Coproparasitology	Salt	0	N/A						Perrucci et al. (2023)
	Ireland	NIM	91	N/A	Coproparasitology PCR	Sucrose	0	No	Nested	ITS1 rDNA	NN1/NN2; NP1/NP2	0		Stuart et al. (2013)
	United States	NIM	49	N/A	Coproparasitology PCR	Sucrose	N/A	No	Convectional	pNc5	Np6 plus/Np21 plus	0		Moreno-Torres et al. (2018)
	Spain	NIM	122	Frozen	Coproparasitology	Sucrose	0							Almería et al. (2002)
*Vulpes vulpes Arabica* (Arabian red fox)	Saudi Arabia	EIM	5	Processed immediately	Coproparasitology PCR	Salt	1	No	Convectional	rDNA large-subunit	CR3/CR5	0	*Hammondia heydorni* (+)	Mohammed et al. (2003)

Ref.:

NIM: Natural infection model.

EIM: Experimental infection model.

N: number of samples.

Salt: Salt solution flotation technique.

Sucrose: Sucrose solution flotation technique.

SAF: standard sodium acetate acetic acid formalin technique.

Coprological analysis N (+): number of positive samples with oocysts *Neospora*/*Hammondia* like.

Molecular analysis N (+): number of positive samples with *Neospora caninum* DNA.

*Hammondia heydorni* (+): positive results.

*Hammondia heydorni* (−): negative results.

N/A: no data.

#### 3.1.1 Species and geographic distribution

These studies evaluated ten wild canid species (Table 1), including one case of hybridization between wild dogs and dingoes in Australia, and one red fox subspecies. Geographically, ten studies were conducted in the Americas (Almeida et al., 2019; Dubey et al., 2011; Gondim et al., 2004a, 2018; Klein et al., 2019; Lindsay et al., 1996; Mannise et al., 2021; Moreno-Torres et al., 2018; Soares et al., 2009; Wapenaar et al., 2006), seven in Europe (Almería et al., 2002; Bandelj et al., 2023; Constantin et al., 2011; Hermosilla et al., 2017; Perrucci et al., 2023; Schares et al., 2002; Stuart et al., 2013), two in Oceania (Davidson et al., 2022; King et al., 2010), and one in Asia (Mohammed et al., 2003) (Fig. 2).

**Fig. 2:**
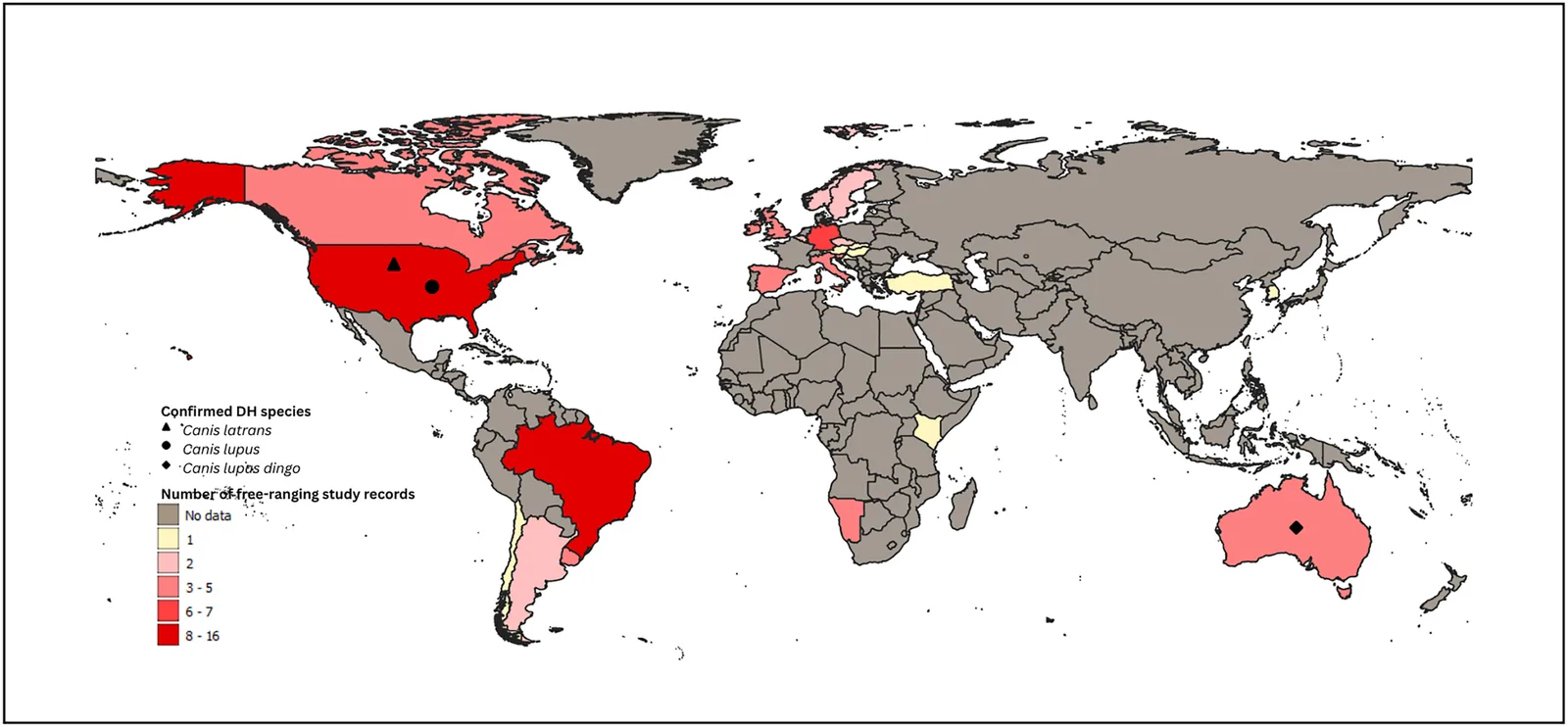
**Geographic distribution of wild canid studies included in the review**. Countries are shaded according to the number of Natural Infection Model (NIM) and Monitoring in Wild Animals (MWA) records identified in the literature. Symbols indicate the three wild canid species confirmed as definitive hosts of *Neospora caninum*: *Canis latrans* (●), *Canis lupus* (▲), and *Canis lupus dingo* (◆).

#### 3.1.2 Study designs: natural infection vs. experimental infection models

The studies were classified into two methodological approaches: Natural Infection Models (NIM), involving the collection of faecal samples from the environment or deceased animals (e.g., roadkill or hunted animals); and Experimental Infection Models (EIM), where captive canids were fed *N. caninum* infected tissues under controlled conditions with daily faecal collection was performed.

• NIM studies (14 papers): Faecal samples were obtained from roadkill or hunted animals in six studies (Almería et al., 2002; Bandelj et al., 2023; Constantin et al., 2011; Dubey et al., 2011; Stuart et al., 2013; Wapenaar et al., 2006), from captive animals in one (Almeida et al., 2019), and directly from the environment using transects in five (Hermosilla et al., 2017; Klein et al., 2019; Mannise et al., 2021; Moreno-Torres et al., 2018; Perrucci et al., 2023). Additionally, one study used wildlife sniffer dogs (Davidson et al., 2022) and another obtained the samples from a canid treated in a veterinary hospital (Gondim et al., 2018).
• EIM studies (6 papers): these articles focused on animals raised in captivity, including fox farms, zoos, or conservation centers (Gondim et al., 2004a; King et al., 2010; Lindsay et al., 1996; Mohammed et al., 2003; Schares et al., 2002; Soares et al., 2009). The age of the animals ranged from 20 days (Lindsay et al., 1996), between 2 and 6 months (Gondim et al., 2004a; King et al., 2010; Mohammed et al., 2003; Soares et al., 2009), up to one year old (Schares et al., 2002). In just two designs, domestic dogs were used as positive infection control (King et al., 2010; Schares et al., 2002). Prior to inoculation, all animals were confirmed as "non-infected" via serological and coprological techniques. Infection was induced by feeding the individuals with tissues from mice (Lindsay et al., 1996), sheep/goats (Schares et al., 2002), gazelles (Mohammed et al., 2003), or bovines (Gondim et al., 2004a; King et al., 2010; Soares et al., 2009).

#### 3.1.3 Methodological analysis for DH determination

• *Sample size and matrix type:*

Sample sizes in NIM papers varied greatly, ranging from 1 to 2191 faecal samples, while EIM studies were more restricted, ranging from three to eight individuals (Table 1). The most frequently monitored animal species was de red fox (*Vulpes vulpes*) with near 2900 samples analysed (Table 1).

Faecal samples were the primary biological matrix used to investigate oocyst shedding in both NIM and EIM (Almeida et al., 2019; Constantin et al., 2011; Davidson et al., 2022; Dubey et al., 2011; Gondim et al., 2018; Hermosilla et al., 2017; Klein et al., 2019; Mannise et al., 2021; Mohammed et al., 2003; Moreno-Torres et al., 2018; Perrucci et al., 2023; Schares et al., 2002; Stuart et al., 2013). In addition to faecal examination, some authors also collected blood for serological analyses (Wapenaar et al., 2006; Gondim et al., 2004a), whereas others included tissue collection for histopathological or molecular investigation (Almería et al., 2002; Bandelj et al., 2023). In three EIM studies, animals were euthanized at the end of the experimental period to allow detailed tissue analyses (King et al., 2010; Lindsay et al., 1996; Soares et al., 2009). Furthermore, EIM protocols generally included daily faecal collection over periods of approximately 25-33 days to monitor the dynamics of oocyst shedding.

• *Sample preservation:*

Different preservation protocols were applied for faecal sample across studies. The most common approach was preserving the samples by freezing, refrigeration, or storage in alcohol prior to analysis (Almeida et al., 2019; Almería et al., 2002; Bandelj et al., 2023; Constantin et al., 2011; Davidson et al., 2022; Hermosilla et al., 2017; Klein et al., 2019; Lindsay et al., 1996; Mannise et al., 2021; Perrucci et al., 2023). Freezing was the most frequently reported preservation method in natural infection studies, whereas refrigeration and alcohol preservation were less commonly employed. Seven studies performed an immediate processing following sample collection, particularly in experimental infection approach (Dubey et al., 2011; Gondim et al., 2004a, 2018; Mohammed et al., 2003; Schares et al., 2002; Soares et al., 2009; Wapenaar et al., 2006). Notably, three studies did not provide detailed information regarding the preservation procedures used for faecal samples (King et al., 2010; Moreno-Torres et al., 2018; Stuart et al., 2013).

• *Diagnostic techniques:*

The studies primarily relied on coproparasitological techniques for oocyst detection. Sucrose flotation was the most frequent method used in both NIM (Almeida et al., 2019; Almería et al., 2002; Constantin et al., 2011; Dubey et al., 2011; Gondim et al., 2018; Moreno-Torres et al., 2018; Stuart et al., 2013; Wapenaar et al., 2006) and EIM (Gondim et al., 2004a; Lindsay et al., 1996; Schares et al., 2002; Soares et al., 2009). Other techniques included Willis flotation (Perrucci et al., 2023; King et al., 2010; Mohammed et al., 2003), Ritchie sedimentation (Mannise et al., 2021), and sodium acetate-acetic acid-formalin (SAF) fixation and sedimentation (Hermosilla et al., 2017).

The standard purification process for oocyst concentration comprised sucrose flotation, collection of the upper layer, and subsequent washing steps with water. Oocysts were then allowed to sporulate at controlled temperature using either 2% potassium dichromate (K_2_Cr_2_O_7_) (Schares et al., 2002) or 2% sulphuric acid (H_2_SO_4_) (Dubey et al., 2011). Conversely, Gondim et al. (2004a), initially conducted the sporulation process using 2% sulphuric acid (H_2_SO_4_), followed by sucrose flotation and a final water-wash step.

Molecular analysis was commonly employed, either as a standalone diagnostic tool (Bandelj et al., 2023; Davidson et al., 2022; Klein et al., 2019), or in combination with coprological techniques (Almeida et al., 2019; Constantin et al., 2011; Dubey et al., 2011; Gondim et al., 2004a, 2018; King et al., 2010; Mannise et al., 2021; Mohammed et al., 2003; Moreno-Torres et al., 2018; Schares et al., 2002; Soares et al., 2009; Stuart et al., 2013; Wapenaar et al., 2006). These molecular diagnostics included conventional, nested, semi-nested, and different real-time PCRs, targeting the ITS1 and pNc5 genomic regions, with Np21+/Np6+ being the most frequently used primers set. Furthermore, specialized confirmation methods such as mice-bioassay (Dubey et al., 2011; Lindsay et al., 1996) and cell culture (Dubey et al., 2011) were used.

• *Definitive Host Confirmation:*

The role of wild canids as DH of *N. caninum* was confirmed via NIM for *C. lupus* (Dubey et al., 2011), and via EIM for *C. latrans* (Gondim et al., 2004a) and *C. lupus dingo* (King et al., 2010). These diagnoses were based on the microscopic visualization of *Neospora-like* oocysts, subsequently confirmed by molecular analysis. In one grey wolf, approximately 10,000 oocysts were estimated from a positive faecal sample (Dubey al., 2011). One experimentally infected coyote shed approximately 500 oocysts over a 2-day period (Gondim et al., 2004a), whereas one dingo shed an estimated 1810 oocysts over 3 days (King et al., 2010).

In contrast, for several NIM studies, *C. aureus* and *C. lupus* (Bandelj et al., 2023), *C. latrans* (Klein et al., 2019), *C. lupus dingo* hybrids (Davidson et al., 2022), *C. thous* (Almeida et al., 2019), *L. gymnocercus* (Mannise et al., 2021), samples yielded positive results exclusively for the presence of *N. caninum* DNA, with no oocyst shedding visualized under microscopy. Furthermore, while Wapenaar et al. (2006) reported *Neospora*-like oocysts in *C. latrans* and *V. vulpes,* subsequent sequencing showed only 95-99% identity to *N. caninum*. Detailed results of each study are provided in Table 1.

The main findings obtained from studies assessing definitive host status are summarized in Fig. 3. Although 20 studies investigated faecal shedding of *N. caninum* in wild canids, evidence progressively decreased from reports of *Neospora*-like oocysts to molecularly confirmed findings, resulting in confirmation of only three *Canis* species as DH.

**Fig. 3:**
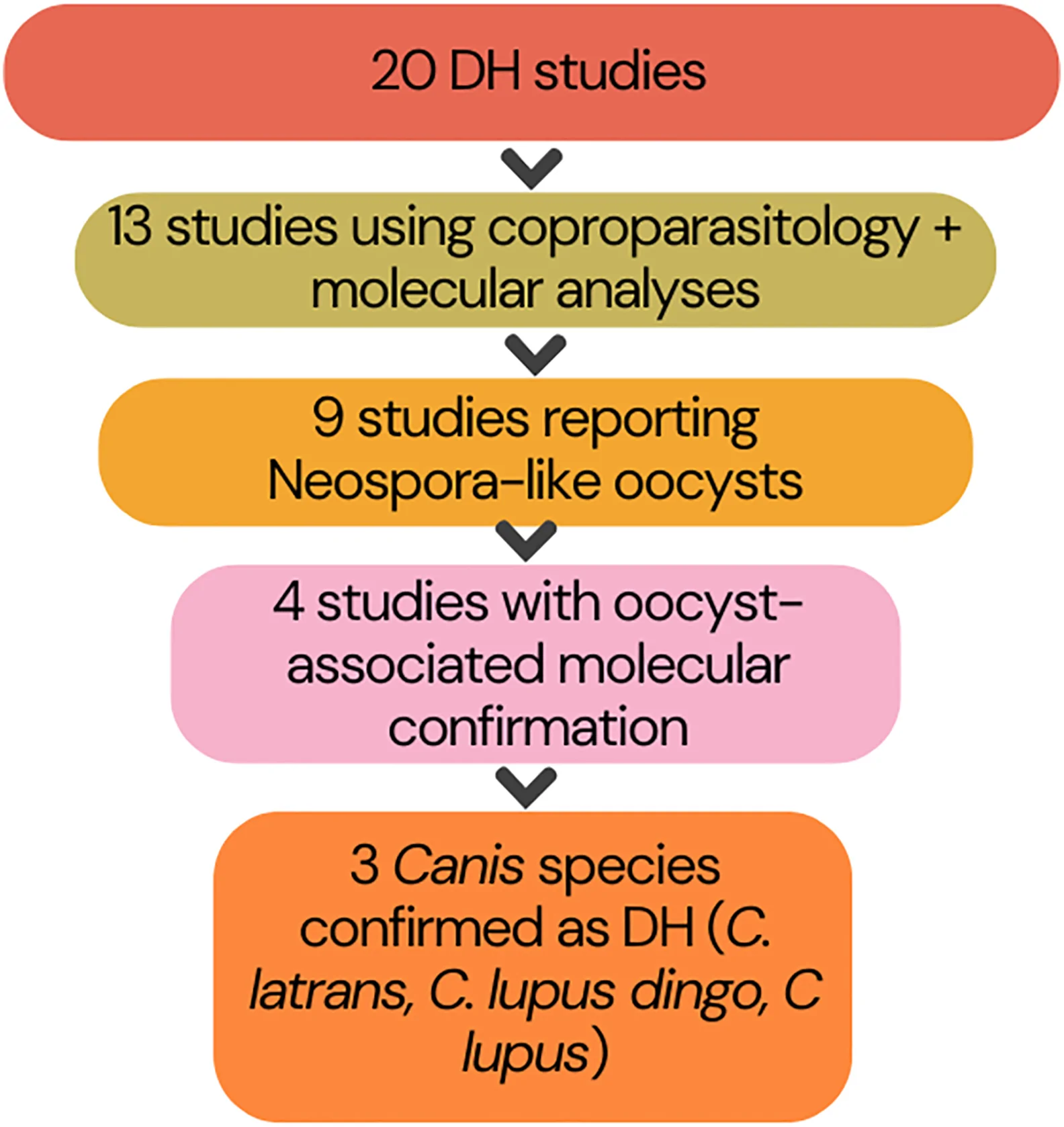
**Overview of evidence supporting the definitive host role of wild canids for *Neospora caninum*.** Twenty studies evaluated the potential role of wild canids as DH of *N. caninum*. Among them, 13 studies combined coproparasitological and molecular approaches, only nine reported the detection of *Neospora*-like oocysts. Four studies provided molecular confirmation associated with the oocysts, finally providing evidence that three species of the genus Canis (*C. latrans, C. lupus dingo and C. lupus*) were definitive hosts.

### 3.2 Wild canids exposed to *N. caninum*

The assessment of *N. caninum* exposure and infection in wild canids across the world was reported by 67 research papers and detailed in Table 2, Table 3.

**Table 2:** Description of studies analyzing wild canids exposed to *N. caninum*. Serological analysis.

Canid sp.	Country	Study	N	Analysis	Cut off	Positive		Observation	Author
						N(+)	%		
*Alopex lagopus* (Artic fox)	China	MCA	103	c-ELISA	N/A	28	27.2		Yu et al. (2009)
*Canis aureus* (Golden Jackal)	Israel	MWA	189	IFAT	1:200	4	2.1	Confirmation by WB: 2/4	Mazuz et al. (2018)
				MAT	1:200	29	15.3	Confirmation by WB: 6/29	
		MWA	114	IFAT	1:50	2	1.8		Steinman et al. (2006)
*Canis latrans* (Coyote)	Canada	MWA	185	IFAT	1:50	0	0		Wapenaar et al. (2006)
	United States	MWA	52	IFAT	1:25	5	10		Lindsay et al. (1996)
		MCA	3	IFAT	1:25	3	100		
		MCA	4	IFAT	1:50	4	100		Gondim et al. (2004a)
		MWA	113	IFAT	1:50	12	11		Gondim et al. (2004b)
		MWA	12	IFAT	1:100	2	16.7		Stieve et al. (2010)
		MWA	394	i-ELISA	1:10	71	18		Bevins et al. (2013)
*Canis lupus* (Wolf)	Brazil	MCA	3	IFAT	1:25	2	66.7		André et al. (2010)
	Czech Republic and Slovakia	MCA	10	IFAT	1:40	2	20		Sedlák and Bártová (2006)
	Israel	MWA	9	IFAT	1:400	1	11.1		Steinman et al. (2006)
	Slovenia	MCA	1	i-ELISA	N/A	0	0		Kvapil et al. (2021)
		MCA	6	i-ELISA	N/A	2	33.3		Račka et al. (2023)
	Spain	MWA	28	c-ELISA	N/A	7	25	Confirmation by IFAT: 0/7 Confirmation by NAT: 6/7	Sobrino et al. (2008)
	United States	MWA	164	IFAT	1:50	64	39		Gondim et al. (2004b)
		MWA	122	NAT	1:40	4	3.3		Dubey and Thulliez (2005)
		MWA	285	NAT	1:50	173	60.7		Carstensen et al. (2017)
		MWA	324	IFAT	1:100	29	9		Stieve et al. (2010)
		MWA	100	IFAT	1:50	0	0		Watts and Benson (2016)
	Sweden and Norway	MWA	109	Iscom-ELISA	1:100	4	3.7		Björkman et al. (2010)
*Canis lupus dingo* (Dingo)	Australia	MWA	52	IFAT	1:50	14	27		Barber et al. (1997)
		MWA	117	IFAT	1:50	1	0.9		
		MCA	4	c-ELISA	N/A	2	50		King et al. (2010)
	Czech Republic and Slovakia	MCA	2	IFAT	1:40	0	0		Sedlák and Bártová (2006)
*Canis mesomelas* (Black-backed jackal)	Namibia	MWA	39	i-ELISA (in-house)	1:100	3	7.7	Confirmation by WB: 1/3	Seltmann et al. (2020)
*Cerdocyon thous* (Crab-eating fox)	Brazil	MCA	39	IFAT	1:25	13	33.3		André et al. (2010)
		MCA	25	NAT	1:50	9	36		Almeida et al. (2018)
		MWA	15	IFAT	1:50	4	26.6	Multiple serological tests (no confirmatory assay)	Cañón-Franco et al. (2004)
				NAT	1:40	1	6.6		
		MWA	10	IFAT	1:40	0	0		de Almeida Curi et al. (2010)
		MCA	25	IFAT	1:50	9	36		Mattos et al. (2008)
		MWA	30	c-ELISA	N/A	1	3	OD > 0.5 (+)	Padilha et al. (2021)
		MWA	7	IFAT	1:50	0	0		Proença et al. (2013)
*Chrysocyon brachyurus* (Maned wolf)	Argentina	MWA	3	IFAT	1:200	0	0		Orozco et al. (2014)
	Brazil	MCA	21	IFAT	1:25	5	23.8		André et al. (2010)
		MWA	7	IFAT	1:40	0	0		de Almeida Curi et al. (2010)
		MWA	10	IFAT	1:50	0	0		de Almeida Curi et al. (2012)
		MWA	17	IFAT	1:50	0	0		de Oliveira et al. (2016)
		MCA	14	IFAT	1:50	4	28.6		Mattos et al. (2008)
		MWA	3	IFAT	1:50	0	0		Proença et al. (2013)
		MCA	59	IFAT	1:25	5	8.5		Silva et al. (2005)
		MCA	59	IFAT	1:25	5	8.5		Vitaliano et al. (2004)
	Czech Republic and Slovakia	MCA	6	IFAT	1:40	1	16.6		Sedlák and Bártová (2006)
*Lycalopex fulvipes* (Darwin fox)	Chile	MWA	26	ELISA	N/A	0	0		Hidalgo-Hermoso et al. (2022)
*Lycalopex gymnocercus* Pampas fox)	Brazil	MWA	12	IFAT	1:50	5	41.6	Multiple serological tests (no confirmatory assay)	Cañón-Franco et al. (2004)
				NAT	1:40	5	41.6		
		MCA	5	IFAT	1:50	3	60		Mattos et al. (2008)
		MWA	22	c-ELISA	N/A	2	9		Padilha et al. (2021)
*Lycalopex vetulus* (Hoary fox)	Brazil	MCA	7	IFAT	1:25	4	57.1		André et al. (2010)
		MWA	30	IFAT	1:50	0	0	Multiple serological tests (no confirmatory assay)	Cañón-Franco et al. (2004)
				NAT	1:40	0	0		
		MWA	2	IFAT	1:40	0	0		de Almeida Curi et al. (2010)
*Lycaon pictus* (African wild dog)	Namibia	MWA	7	i-ELISA (in-house)	1:100	0	0	Confirmation by WB: 0/7	Seltmann et al. (2020)
	Kenia	MWA	87	IFAT	1:40	45	52		Woodroffe et al. (2012)
	Czech Republic and Slovakia	MCA	9	IFAT	1:40	0	0		Sedlák and Bártová (2006)
*Nyctereutes procyonoides koreensis* (Korean raccoon dog)	Korea	MWA	26	NAT	1:512	6	23		Kim et al. (2003)
*Otocyon megalotis* (Bat-eared fox)	Namibia	MWA	4	i-ELISA (in house)	1:100	0	0	Confirmation by WB: 0/4	Seltmann et al. (2020)
*Speothos venaticus* (Bush dog)	Brazil	MCA	27	IFAT	1:25	16	59.2		André et al. (2010)
		MCA	6	IFAT	1:50	2	33.3		Mattos et al. (2008)
	Czech Republic and Slovakia	MCA	3	IFAT	1:40	0	0		Sedlák and Bártová (2006)
*Urocyon cinereoargenteus* (Grey fox)	United States	MWA	26	NAT	1:25	4	15.4		Lindsay et al. (2001)
*Vulpes vulpes* (Red fox)	Austria	MWA	94	IFAT	1:50	0	0		Wanha et al. (2005)
	Belgium	MWA	123	IFAT	1:64	21	17		Buxton et al. (1997)
	Canada	MWA	271	IFAT	1:50	0	0		Wapenaar et al. (2006)
	Czech Republic	MWA	80	IFAT	1:25	3	3.8	Multiple serological tests (no confirmatory assay)	Bártová et al. (2016)
				c-ELISA	N/A	2	2.5		
	Hungary	MWA	337	Iscom-ELISA (in house)	1:100	5	1.5		Jakubek et al. (2007)
	Israel	MWA	75	IFAT	1:200	1	1.3	Confirmation by WB: 1/1	Mazuz et al. (2018)
				MAT	1:200	9	12	Confirmation by WB: 1/9	
		MWA	24	IFAT	1:400	1	4.2		Steinman et al. (2006)
	Ireland	MWA	206	IFAT	1:100	6	2.9	Pleural fluid	Murphy et al. (2007)
		MWA	101	IFAT	1:40	1	0.9		Stuart et al. (2013)
		MWA	70	IFAT	1:20	1	1.4		Wolfe et al. (2001)
	Italy	MWA	10	FASTest	N/A	0	0	Immunochromatography	Pacini et al. (2023)
	Slovakia	MWA	177	c-ELISA	N/A	69	38.9	OD ≥ 0.3 (+).	Reiterová et al. (2016)
		MWA	126	c-ELISA	N/A	11	8.7	Muscle fluid OD ≥ 0.3 (+).	
	Spain	MWA	53	NAT	1:40	37	69.8		Marco et al. (2008)
		MWA	95	c-ELISA	N/A	11	11.6	Confirmation by IFAT: 2/9 Confirmation by NAT: 2/6	Sobrino et al. (2008)
	Sweden	MWA	221	Iscom-ELISA (in house)	N/A	0	0		Jakubek et al. (2001)
	United States	MWA	9	IFAT	1:100	0	0		Stieve et al. (2010)
	United Kingdom	MWA	54	IFAT	1:50	1	2		Barber et al. (1997)
		MWA	549	IFAT	1:16	5	0.9	Pulmonar fluid	Hamilton et al. (2005)
*Vulpes zerda* (Fennec)	Czech Republic and Slovakia	MCA	2	IFAT	1:40	2	100		Sedlák and Bártová (2006)

Ref:

N: Number of samples.

MCA: Monitoring in Captive Animals.

MWA: Monitoring in Wild Animals.

N (+): number of positive samples at serological test.

%: positive results as a percentage.

N/A: No data.

WB: Westernblot antibody test.

OD: Optical density.

**Table 3:** Description of studies analyzing wild canids exposed to *N. caninum*. Tissues’ analysis.

′Canid sp.	Country	Type of tissue	Analysis	N	HP	IHC	PCR						Author
					% (+)	% (+)	Type of PCR	Target	Primers	N (+)	% (+)	Observations	
*Alopex lagopus* (Arctic fox)	China	Brain, muscle, lung, heart liver	HP, IHC, PCR	28	17.9	17.9	Seminested	pNc5	Np21/Np6; Np6/Np7	4	14.3		Yu et al. (2009)
	Norway	Brain, lung, liver	IHC	3		0							Sørensen et al. (2005)
*Canis aureus* (Golden Jackal)	Slovenia	N/A	PCR	9			Real time	pNc5	Np1/Np2	1	11.1		Bandelj et al. (2023)
*Canis latrans* (Coyote)	United States	Brain	Mice-bioassay	3								Bioassay (−)	Lindsay et al. (1996)
*Canis lupus* (Wolf)	United States	Brain, heart	PCR, Mice-bioassay, Cell-culture	109			Convencional	pNc5	Np21plus/Np6plus	2	1.8	Bioassay (+), Cell-culture (+)	Dubey et al. (2014)
	Slovenia	N/A	PCR	31			Real time	pNc5	Np1/Np2	3	9.6		Bandelj et al. (2023)
*Cerdocyon thous* (Crab-eating fox)	Brazil	Brain, muscle	PCR	4			Seminested	pNc5	Np4/Np7,Np6/Np7	0	0		Soares et al. (2009)
		Brain	PCR	5			Nested	18S rDNA Apicomplexa		0	0		Balbino et al. (2022)
	Uruguay	Muscle, heart, liver, kidney, skin	PCR	55			Real time TaqMan		Nc-UY forward, Nc-UY reverse	0	0		Mannise et al. (2021)
*Lycalopex gymnocercus* (Pampas fox)	Argentina	Brain, muscle	HP, IHC, PCR	41	4	0	Convencional	pNc5	Np21plus/Np6plus	23	56.1		Scioscia et al. (2022)
							Nested	ITS1 rDNA	NP1/NP2	17	41.5		
	Uruguay	Muscle, heart, liver, kidney, skin	PCR	46			Real time TaqMan		Nc-UY forward, Nc-UY reverse	1	2.2	(+) skin	Mannise et al. (2021)
*Lycalopex vetulus* (Hoary fox)	Brazil	Brain	PCR	49			Convencional	pNc5	Np21/Np6	6	12.2		Nascimento et al. (2015)
*Nyctereutes procyonoides* (Racoon dog)	Germany	Brain	PCR	34			Real time	pNc5	Np21 plus/Np6 plus	0	0		Höche et al. (2022)
		Brain	HP, IHC	10	3	0							Lempp et al. (2017)
*Vulpes vulpes* (Red fox)	Belgium	Brain	PCR	304			Multiplex quantitative real-time PCR	pNc5	Np21/Np6	20	6.6	Probes NP	Hůrková and Modrý (2006)
								ITS1 rDNA	NF1/NR2				
	Czech Republic	Brain	PCR	152			Convencional	pNc5	Np21 plus/Np6 plus	7	4.6		Hůrková and Modrý (2006)
		Brain	PCR	100			Convencional	pNc5	Np21 plus/Np6 plus	0	0		Lukášová et al. (2018)
	Germany	Brain	PCR	769			Real time TaqMan	pNc5	Np21plus/Np6plus	0	0		Constantin et al. (2011)
		Brain	HP, IHC	9	0	0							Lempp et al. (2017)
		Brain	PCR	860			Real time	pNc5	Np21 plus/Np6 plus	0	0		Höche et al. (2022)
	Ireland	Brain	HP, PCR	148	22.3		Convencional	pNc5	Np21/Np6	0	0		Murphy et al. (2007)
		Brain	PCR	151			Nested	ITS1 rDNA	NN1/NN2,NP1/NP2	9	6		Stuart et al. (2013)
	Italy	Brain	PCR	71			Convencional	pNc5	Np21/Np6 plus	0	0		Ebani et al. (2022)
		Brain	PCR	17			Convencional	pNc5	Np21 plus/Np6 plus	6	35.3	(+) Adults females and fetus 100% congenital transmission	Zanet et al. (2023)
	Scotland-England	Brain	PCR	83			Nested	ITS1 rDNA	NN1/NN2,NP1/NP2	4	4.8		Bartley et al. (2013)
	Slovakia	Brain, muscle, blood	PCR	177			Convencional	pNc5	Np21/Np6	19	10.7		Reiterová et al. (2016)
		Brain, muscle	PCR	126			Convencional	pNc5	Np21/Np6	3	2.4		
	Spain	Brain	PCR	122			Convencional	pNc5	Np21 plus/Np6 plus	13	10.7		Almería et al. (2002)
	Turkiye	Brain	PCR	3			Convencional	pNc5	Np21/Np6	1	33.3		Erol et al. (2023)

Ref:

N: number of samples.

HP: Histopathology analysis.

IHC: Inmunohistochemistry analysis.

%(+): percentage of positive samples to *Neospora caninum*.

N (+): number of *Neospora caninum* positive samples.

N/A: no data.

#### 3.2.1 Species and geographic distribution

A total of 19 species of wild canids were evaluated, including one subspecies of raccoon dog. The geographic distribution of these studies showed a predominant focus on Europe and the Americas (Fig. 2), which together accounted for nearly 90% (59/67) of the selected articles (Table 2, Table 3). Europe: Thirty of the 67 articles were conducted in this region. Research has been extensive in countries such as Italy, Spain, and the Czech Republic, with the red fox (*V. vulpes*) being the most frequently sampled species (Almería et al., 2002; Barber et al., 1997; Bartley et al., 2013; Bártová et al., 2016; Buxton et al., 1997; Constantin et al., 2011; De Craeye et al., 2011; Ebani et al., 2022; Hamilton et al., 2005; Höche et al., 2022; Hůrková and Modrý, 2006; Jakubek et al., 2001, 2007; Lempp et al., 2017; Lukášová et al., 2018; Marco et al., 2008; Murphy et al., 2007; Pacini et al., 2023; Račka et al., 2023; Reiterová et al., 2016; Sobrino et al., 2008; Stuart et al., 2013; Wanha et al., 2005; Wolfe et al., 2001; Zanet et al., 2023).

The Americas: Twenty-nine of the 67 studies were carried out on this continent. These studies were distributed between North America, focusing primarily on *C*. *latrans* and *C. lupus* (Bevins et al., 2013; Carstensen et al., 2017; Dubey et al., 2014; Dubey and Thulliez, 2005; Gondim et al., 2004a, 2004b; Lindsay et al., 1996; Stieve et al., 2010; Wapenaar et al., 2006; Watts and Benson, 2016), and South America, where studies frequently targeted *C. thous* and *Lycalopex* spp. (Almeida et al., 2018; André et al., 2010; Balbino et al., 2022; Cañón-Franco et al., 2004; de Almeida Curi et al., 2010; Hidalgo-Hermoso et al., 2022; Mannise et al., 2021; Mattos et al., 2008; Nascimento et al., 2015; Padilha et al., 2021; Proença et al., 2013; Scioscia et al., 2022; Soares et al., 2009).

Other Regions: In contrast, only five studies were identified in Asia (Erol et al., 2023; Kim et al., 2003; Mazuz et al., 2018; Steinman et al., 2006; Yu et al., 2009), two in Oceania (Barber et al., 1997; King et al., 2010), and two in Africa (Seltmann et al., 2020; Woodroffe et al., 2012), highlighting a significant knowledge gap regarding the exposure of wild canids in these regions.

Overall, across the 19 wild canid species assessed, red fox (*V. vulpes*) had the highest number of documented reports, with a total of 28 publications (Table 2, Table 3).

#### 3.2.2 Monitoring in wild (MWA) vs. Captive (MCA) animals

The screening identified two categories of studies assessing *N. caninum* exposure in wild canids: Monitoring in Wild Animals (MWA), comprising 56 articles involving captured or deceased animals in the wild; and Monitoring in Captive Animals (MCA), with 13 works where samples were collected from zoo animals, private collections or commercial breeding farms, including experimental infection studies.

• Research goals

The primary aim of most surveys (47/67) was to detect anti-*N. caninum* antibodies, including studies in the MWA category (Barber et al., 1997; Bártová et al., 2016; Bevins et al., 2013; Björkman et al., 2010; Buxton et al., 1997; Cañón-Franco et al., 2004; Carstensen et al., 2017; de Almeida Curi et al., 2010, 2012; de Oliveira et al., 2016; Dubey and Thulliez, 2005; Gondim et al., 2004b; Hamilton et al., 2005; Hidalgo-Hermoso et al., 2022; Jakubek et al., 2001, 2007; Kim et al., 2003; Lindsay et al., 1996, 2001; Marco et al., 2008; Mazuz et al., 2018; Murphy et al., 2007; Orozco et al., 2014; Pacini et al., 2023; Padilha et al., 2021; Proença et al., 2013; Seltmann et al., 2020; Sobrino et al., 2008; Steinman et al., 2006; Stieve et al., 2010; Stuart et al., 2013; Wanha et al., 2005; Wapenaar et al., 2006; Watts and Benson, 2016; Wolfe et al., 2001; Woodroffe et al., 2012) and the MCA category (Almeida et al., 2018; André et al., 2010; Gondim et al., 2004a; King et al., 2010; Kvapil et al., 2021; Mattos et al., 2008; Račka et al., 2023; Sedlák and Bártová, 2006; Silva et al., 2005; Vitaliano et al., 2004; Yu et al., 2009). Additionally, 22 surveys focused on identifying tissue cysts (o stages) via histology and/or DNA detection in wild species, comprising 20 MWA studies (Almería et al., 2002; Balbino et al., 2022; Bandelj et al., 2023; Bartley et al., 2013; Constantin et al., 2011; De Craeye et al., 2011; Dubey et al., 2014; Ebani et al., 2022; Erol et al., 2023; Höche et al., 2022; Hůrková and Modrý, 2006; Lempp et al., 2017; Lukášová et al., 2018; Mannise et al., 2021; Murphy et al., 2007; Nascimento et al., 2015; Scioscia et al., 2022; Sørensen et al., 2005; Stuart et al., 2013; Zanet et al., 2023), and two MCA works (Soares et al., 2009; Yu et al., 2009). Only two studies integrated both approaches by assessing antibodies and tissue cysts o stages concurrently, one MWA (Reiterová et al., 2016) and one MCA (Lindsay et al., 1996).

• Sampling and Study Design

The methodology for obtaining samples and the scale of the studies varied between the two monitoring categories:

• In MWA: Sample sizes ranged from 2 to 860 individuals. Twenty-six papers involved the capture of live animals for blood sampling and their subsequent release (Bártová et al., 2016; Bevins et al., 2013; Björkman et al., 2010; Buxton et al., 1997; Cañón-Franco et al., 2004; Carstensen et al., 2017; de Almeida Curi et al., 2010, 2012; de Oliveira et al., 2016; Dubey and Thulliez, 2005; Gondim et al., 2004b; Hidalgo-Hermoso et al., 2022; Jakubek et al., 2001; Kim et al., 2003; Lindsay et al., 1996, 2001; Orozco et al., 2014; Pacini et al., 2023; Padilha et al., 2021; Proença et al., 2013; Seltmann et al., 2020; Sobrino et al., 2008; Steinman et al., 2006; Stieve et al., 2010; Watts and Benson, 2016; Woodroffe et al., 2012). In contrast, thirty-two studies obtained samples from roadkilled or hunted animals (Almería et al., 2002; Balbino et al., 2022; Bandelj et al., 2023; Barber et al., 1997; Bartley et al., 2013; Carstensen et al., 2017; Constantin et al., 2011; De Craeye et al., 2011; Dubey et al., 2014; Ebani et al., 2022; Erol et al., 2023; Hamilton et al., 2005; Höche et al., 2022; Hůrková and Modrý, 2006; Jakubek et al., 2007; Lempp et al., 2017; Lukášová et al., 2018; Mannise et al., 2021; Marco et al., 2008; Mazuz et al., 2018; Murphy et al., 2007; Nascimento et al., 2015; Reiterová et al., 2016; Scioscia et al., 2022; Seltmann et al., 2020; Sobrino et al., 2008; Sørensen et al., 2005; Stuart et al., 2013; Wanha et al., 2005; Wapenaar et al., 2006; Wolfe et al., 2001; Zanet et al., 2023). Regarding sample matrices, blood or serum was the primary source for serology (n = 35), although some studies utilized alternative fluids such as pleural, lung, or muscle fluids.
• In MCA: Sample sizes were generally smaller, ranging from 1 to 103. Samples were obtained from animals kept in captivity in nine papers (Almeida et al., 2018; André et al., 2010; Kvapil et al., 2021; Mattos et al., 2008; Račka et al., 2023; Sedlák and Bártová, 2006; Silva et al., 2005; Vitaliano et al., 2004; Yu et al., 2009), while four papers focused on experimental infection models or bioassays conducted under controlled conditions (Gondim et al., 2004a; King et al., 2010; Lindsay et al., 1996; Soares et al., 2009). Blood was the exclusive sample type used for serological analysis in this category (n = 12).

Across both categories, tissue analysis to identify the parasite's presence included the examination of the brain, muscle, heart, lung, and liver (Table 3). Details regarding the specific fluids and tissues analysed for each study are summarized in Table 2, Table 3

#### 3.2.3 Diagnostic methods for exposure analysis

##### 3.2.3.1 Serological analysis

Several serological tests were used, either alone or in combination, to investigate exposure to *N. caninum* in wild canids. The results of the serological test are summarized in Table 2.

• Indirect Immunofluorescence Antibody test (IFAT): was the most frequently employed method, being used in 65% of the serological studies both in MWA (Barber et al., 1997; Bártová et al., 2016; Buxton et al., 1997; Cañón-Franco et al., 2004; de Almeida Curi et al., 2010, 2012; de Oliveira et al., 2016; Gondim et al., 2004b; Hamilton et al., 2005; Mazuz et al., 2018; Murphy et al., 2007; Orozco et al., 2014; Proença et al., 2013; Sobrino et al., 2008; Steinman et al., 2006; Stieve et al., 2010; Stuart et al., 2013; Wanha et al., 2005; Wapenaar et al., 2006; Watts and Benson, 2016; Wolfe et al., 2001; Woodroffe et al., 2012) and MCA settings (André et al., 2010; Gondim et al., 2004a; Lindsay et al., 1996; Mattos et al., 2008; Sedlák and Bártová, 2006; Silva et al., 2005; Vitaliano et al., 2004). Most of them applied a cut-off titter of 1:50 and commonly used the Nc-1 strain as antigen, although cut-off values varied considerably among studies, ranging from 1:25 to 1:400 (Table 2).
• Enzyme-Linked Immunosorbent Assay (ELISA): were the second most frequently used serological approach (32% of studies). Several ELISA were reported, including commercial competitive ELISAs (c-ELISA), indirect ELISAs (i-ELISA), iscom-ELISAs, and other in house assays, both in MWA (Bártová et al., 2016; Bevins et al., 2013; Björkman et al., 2010; Hidalgo-Hermoso et al., 2022; E.-B. Jakubek et al., 2001; E. B. Jakubek et al., 2007; Padilha et al., 2021; Račka et al., 2023; Reiterová et al., 2016; Seltmann et al., 2020; Sobrino et al., 2008) and MCA studies. Consequently, substantial methodological variability was observed among studies employing ELISA-based technique.
• Other techniques: were less frequently used and included the *Neospora* Agglutination test (NAT) in MWA studies (Cañón-Franco et al., 2004; Carstensen et al., 2017; Dubey and Thulliez, 2005; Kim et al., 2003; Lindsay et al., 2001; Marco et al., 2008; Sobrino et al., 2008) and one MCA (Almeida et al., 2018), Modified Agglutination test (MAT) (Mazuz et al., 2018), Western Blot (WB) (Mazuz et al., 2018; Seltmann et al., 2020), and the Immunochromatographic FASTest (Pacini et al., 2023). While NAT and MAT were primarily used as alternative screening tools (Cañón-Franco et al., 2004; Sobrino et al., 2008), the WB were generally employed as confirmatory methods (Mazuz et al., 2018; Seltmann et al., 2020).

Most studies relied on a single serological assay to infer exposure to *N. caninum*, whereas only a limited number incorporated multiple serological approaches or confirmatory test. Therefore, the reliability of the serological test varied between studies and should be interpreted with caution, considering the diagnostic strategy used.

##### 3.2.3.2 Tissue analysis

Molecular and morphological methods were applied to confirm the presence of the parasite in tissues. Detailed findings for each study are provided in Table 3.

• Morphological and Antigenic Identification: Histopathological examination was conducted in five MWA studies (Dubey et al., 2014; Lempp et al., 2017; Murphy et al., 2007; Scioscia et al., 2022) and in one MCA (Yu et al., 2009), with four of these further employing immunohistochemistry (IHC) to confirm the presence of specific parasitic stages (Lempp et al., 2017; Scioscia et al., 2022; Sørensen et al., 2005; Yu et al., 2009).
• Molecular detection: Parasite DNA was identified in 20 studies using various PCR formats, including conventional, nested, and real-time variants. This approach was applied in both MWA (Almería et al., 2002; Balbino et al., 2022; Bandelj et al., 2023; Bartley et al., 2013; Constantin et al., 2011; De Craeye et al., 2011; Ebani et al., 2022; Erol et al., 2023; Höche et al., 2022; Hůrková and Modrý, 2006; Lukášová et al., 2018; Mannise et al., 2021; Murphy et al., 2007; Nascimento et al., 2015; Reiterová et al., 2016; Scioscia et al., 2022; Stuart et al., 2013; Zanet et al., 2023) and MCA settings (Soares et al., 2009; Yu et al., 2009).
• In vivo confirmation: To assess parasite viability, bioassays were performed in two studies using murine models, one MWA (Dubey et al., 2014) and one MCA (Lindsay et al., 1996).

##### 3.2.3.3 Serological results

Reported seroprevalence estimates ranged from 0% to 100%, with substantial variation among species and study types. Species-specific distributions of seroprevalence estimates, separated according to monitoring category (MWA and MCA), are shown in Fig. 4. In general, a wider range of seroprevalence values was observed in *C. lupus*, *C. latrans*, and *V. vulpes*, whereas higher median values were recorded in *L. gymnocercus*, *L. pictus*, and captive populations of *C. lupus dingo* and *S. venaticus*.

**Fig. 4:**
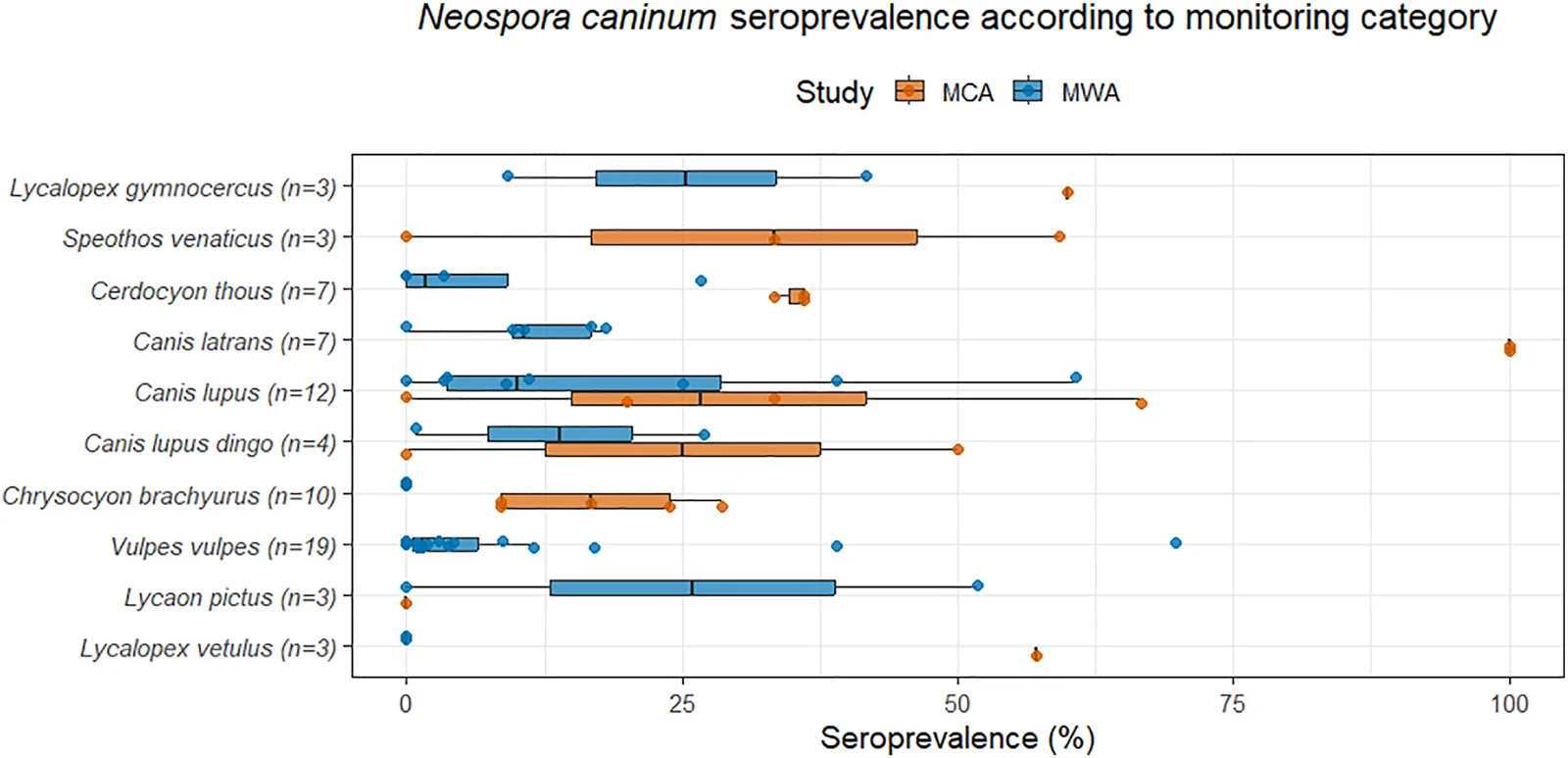
**Distribution of *Neospora caninum* seroprevalence estimates among wild canid species according to monitoring category.** Boxplots show the median, interquartile range (IQR), and variability of seroprevalence estimates reported in the literature. Each point represents an individual seroprevalence record. Monitoring in Wild Animals (MWA, blue) and Monitoring in Captive Animals (MCA, orange) were analysed separately to compare exposure patterns between free-ranging and captive populations. Numbers in parentheses denote the number of seroprevalence records available for each species.

### 3.3 Global distribution of *Neospora caninum* in wild canids

The geographical distribution of studies on free-living wild canids was highly variable, with the majority of records concentrated in Europe, North America and South America, particularly in the United States and Brazil (Fig. 2). By contrast, records from Africa, Asia and Oceania were relatively limited.

## 4 Discussion

### 4.1 Wild canids as definitive hosts

This systematic review analysed 20 studies evaluating the role of wild canids as definitive hosts (DH) of *N. caninum*, using two methodological approaches: Natural Infection Models (NIM) and Experimental Infection Models (EIM). The results obtained have confirmed that the coyote (*C. latrans*) (Gondim et al., 2004a), the dingo (*C. lupus dingo*) (King et al., 2010) under experimental conditions, and the grey wolf (*C. lupus*) (Dubey et al., 2011) under natural conditions have the capacity to shed *N. caninum* oocysts in their faeces (Fig. 3).

Most research (∼80%) is concentrated in the Americas and Europe (Almeida et al., 2019; Almería et al., 2002; Bandelj et al., 2023; Constantin et al., 2011; Dubey et al., 2011; Gondim et al., 2004a, 2018; Hermosilla et al., 2017; Klein et al., 2019; Lindsay et al., 1996; Mannise et al., 2021; Moreno-Torres et al., 2018; Perrucci et al., 2023; Schares et al., 2002; Soares et al., 2009; Stuart et al., 2013; Wapenaar et al., 2006). However, there is a notable absence of research in regions with high biodiversity of wild canids, such as Africa and Southeast Asia, which limits the overall understanding of the sylvatic cycle. Only data on exposure to *N. caninum* have been reported for *L. pictus*, *C. mesomelas* and *O. megalotis* in Africa (Seltmann et al., 2020; Woodroffe et al., 2012), and for *N. procyonoides koreensis* and *A. lagopus* in Asia (Kim et al., 2003; Yu et al., 2009) (Fig. 2).

NIMs reflect the actual natural dynamics of oocyst release in ecosystems, but they present challenges such as confirming the species that originated the faeces, which requires molecular methods (Klein et al., 2019; Mannise et al., 2021; Moreno-Torres et al., 2018) or the use of wildlife detection or sniffer dogs (Davidson et al., 2022). However, the implementation of these tools significantly increases the operational costs of the studies, which may limit the number of samples processed and the feasibility of long-term monitoring in regions with restricted funding. On the other hand, EIMs allow for strict control of the animals' diet and faecal samples, confirming a cause-effect relationship in oocysts elimination (Gondim et al., 2004a; King et al., 2010; Lindsay et al., 1996; Mohammed et al., 2003; Schares et al., 2002; Soares et al., 2009). Despite this, EIMs requires the breeding or capture of individuals a complex task for species that do not reproduce well in captivity, which also raises concerns about bioethical and animal welfare issues. Furthermore, small sample size (3 to 8 individuals) in these studies can compromise the robustness of the results.

A marked heterogeneity in sample preservation was observed; while immediate processing predominated in EIM studies (Gondim et al., 2004a; Mohammed et al., 2003; Schares et al., 2002; Soares et al., 2009), freezing conservation was mostly utilized in NIM studies (Almeida et al., 2019; Almería et al., 2002; Bandelj et al., 2023; Constantin et al., 2011; Davidson et al., 2022; Klein et al., 2019; Perrucci et al., 2023). This variability potentially impacts oocyst integrity and viability and, therefore, the sensitivity of coprological and molecular analyses. The most widely employed coproparasitological technique was sucrose flotation (Almeida et al., 2019; Almería et al., 2002; Constantin et al., 2011; Dubey et al., 2011; Gondim et al., 2004a, 2018; Lindsay et al., 1996; Moreno-Torres et al., 2018; Schares et al., 2002; Soares et al., 2009; Stuart et al., 2013; Wapenaar et al., 2006), presumably due to its high sensitivity and ability to concentrate and preserve the morphology of the oocysts.

An additional aspect that deserves consideration is the intensity of oocyst shedding. Available data indicate that oocyst production in wild canids is generally low, with a coyote shedding approximately 500 oocyst and a dingo shedding about 1810 oocysts during relatively short periods of two to three days (Gondim et al., 2004; King et al., 2010). In a naturally infected grey wolf, approximately 10,000 oocysts were estimated from a positive sample (Dubey et al., 2011). These values are substantially lower than those reported in experimental infections of domestic dogs (Lindsay et al., 1999; Gondim et al., 2002). However, other studies in domestic dogs have documented the excretion of relatively low numbers of oocysts over short shedding periods (Dijkstra et al., 2001; Lindsay et al., 1999; Cavalcante et al., 2011). Nevertheless, the epidemiological significance of oocyst shedding cannot be assessed simply based on the number of oocysts excreted. As reviewed by Gondim and McAllister (2022), experimental infections in cattle demonstrated that exposure to relatively low numbers of viable *N. caninum* oocysts may result in infection and exogenous transplacental transmission. Therefore, even short shedding periods involving comparatively small numbers of oocysts may contribute to environmental contamination and the maintenance of local wild cycles. Coproparasitological and molecular results confirm the DH role in three *Canis* species through experimental infection (Gondim et al., 2004a; King et al., 2010) or detecting natural infected animals (Dubey et al., 2011). In contrast, in several NIM studies, *N. caninum* DNA was detected in faeces from species such as the crab-eating fox (Almeida et al., 2019), the pampas fox (Mannise et al., 2021) and the golden jackal (Bandelj et al., 2023), without microscopic identification of oocysts. These findings may result from environmental contamination or the presence of parasite DNA from alternative sources (e.g., the ingestion of infected prey leading to mechanical passage), rather than true oocyst shedding by species not yet confirmed as DH. Moreover, the sole detection after a nested PCR (Almeida et al., 2019), should be interpreted with caution due to the potential false positive result risk of the technique. To date, only species within the genus *Canis* have been definitively identified as DH of *N. caninum*. This restriction may be linked to specific features of the intestinal environment, particularly host–parasite interactions at the cellular level that enable sexual reproduction of the parasite. However, it remains unclear whether members of other genera or tribes within the Canidae family possess the biological capacity to support the complete life cycle of *N. caninum*. Currently, the impact of wild canids acting as DH and disseminate *N. caninum* oocysts and the consequent environmental contamination appears to be relatively low (Dubey et al., 2011).

### 4.2 Wild canids exposed to *N. caninum*

Studies covering five continents reveal that exposure to *N. caninum* in wild canid populations remains significantly underrepresented in Asia, Africa, and Oceania, a geographic gap that limits the global extrapolation of the parasite's dynamics (Fig. 2). To date, exposure has been assessed in 19 canid species, with the red fox (*V. vulpes*) being the most extensively studied (Almería et al., 2002; Barber et al., 1997; Bartley et al., 2013; Bártová et al., 2016; Buxton et al., 1997; Constantin et al., 2011; De Craeye et al., 2011; Ebani et al., 2022; Erol et al., 2023; Hamilton et al., 2005; Höche et al., 2022; Hůrková and Modrý, 2006; Jakubek et al., 2001, 2007; Lempp et al., 2017; Lukášová et al., 2018; Marco et al., 2008; Mazuz et al., 2018; Murphy et al., 2007; Pacini et al., 2023; Reiterová et al., 2016; Sobrino et al., 2008; Steinman et al., 2006; Stieve et al., 2010; Stuart et al., 2013; Wanha et al., 2005; Wapenaar et al., 2006; Wolfe et al., 2001; Zanet et al., 2023). This research bias is largely influenced by the species-wide Holarctic distribution and the ease of sample access provided by official population control programs. Although the red fox's role as a DH remains unconfirmed (Constantin et al., 2011; Schares et al., 2002), the recurrent detection of antibodies, parasite DNA, and the evidence of vertical transmission suggest that this species may play an important role in maintaining *N. caninum* within the sylvatic cycle.

The comparison between monitoring categories suggests that captive population (MCA) frequently exhibit higher prevalence estimates than free-ranging population (MWA) (Fig. 4). Unlike MCAs, MWA studies offer a more realistic ecological view of *N. caninum* circulation within wildlife communities. Elevated seroprevalence values observed in captive South American canids, including the crab-eating fox (Almeida et al., 2018; André et al., 2010), pampas fox (Mattos et al., 2008), hoary fox (André et al., 2010) and the bush dog (André et al., 2010; Mattos et al., 2008). may be associated with management practices, higher animal densities, restricted environments, or feeding practices that potentially facilitate exposure to *N. caninum*. In contrast, several species investigated under free-ranging condition, such as the maned wolf (de Almeida Curi et al., 2010, 2012; de Oliveira et al., 2016; Orozco et al., 2014; Proença et al., 2013), Darwin's fox (Hidalgo-Hermoso et al., 2022), the African wild dog, and the bat-eared fox (Seltmann et al., 2020), showed no detectable antibodies. However, these findings should be interpreted cautiously, as differences in sampling and diagnostic test performance may account for the apparent absence of exposure.

Serological evidence represented the most common source of information regarding exposure to *N. caninum* in wild canids. IFAT was the predominant serological technique, followed by different ELISAs. Considerable methodological heterogeneity was identified among studies; including differences assay type, cut-off values, and the use or not of confirmatory methods. Such variation may have affected both sensitivity and specificity, which therefore influences the published seroprevalence estimates.

Interpretation of serological findings also requires caution because most assays currently used for *N. caninum* were originally developed and validated for cattle and domestic dogs rather than wild canids species. Consequently, test sensitivity, specificity, and optimal cut-off remain uncertain for many wildlife hosts (Almería, 2013). This limitation is particularly significant when comparing prevalence estimates, since observed differences could reflect methodological variation or epidemiological patterns. Furthermore, only a minority of the studies used confirmatory methods, such as Western blot (Mazuz et al., 2018; Seltmann et al., 2020) or multiple independent serological tests (Cañon Franco et al., 2004; Sobrino et al., 2008), and most were based on the interpretation of a single test. Studies that combine screening and confirmatory methods provide more robust evidence of exposure, as they reduce the uncertainty associated with the specificity of the tests.

It is also important to consider that a seropositive result is interpreted as evidence of previous exposure; however, a seronegative result does not exclude infection. For example, experimental infected domestic dogs have been reported to shed *N. caninum* oocysts without developing detectable antibody responses (Dijkstra et al., 2001; Gondim et al., 2002; Cavalcante et al., 2011), indicating that serological findings alone may underestimate the epidemiological relevance of some hosts. Therefore, serological data should be interpreted primarily as indicators of exposure and considered together with molecular, histopathological, and epidemiological evidence whenever possible.

While serology reflects prior contact with the parasite, molecular and histological studies identify actual infections. This enables the evaluation of key aspects in order to determine the role of the species as IH. *N. caninum* DNA was detected in the CNS and muscle tissue of several asymptomatic species in MWA studies (Almería et al., 2002; Bandelj et al., 2023; Bartley et al., 2013; Dubey et al., 2014; Hůrková and Modrý, 2006; Mannise et al., 2021; Nascimento et al., 2015; Reiterová et al., 2016; Scioscia et al., 2022; Stuart et al., 2013; Zanet et al., 2023). On the other hand, Yu et al. (2009) confirmed infection in Artic foxes with clinical signs, identifying tachyzoite clusters and tissue cysts via immunohistochemistry (IHC) and PCR. However, caution is required in diagnosis: sporadic occurrences of muscle cysts compatible with *N. caninum* have later been identified as *Sarcocystis* spp. (Lempp et al., 2017).

A major highlight of our review is the evidence for the pampas fox as a competent IH (Scioscia et al., 2022) and the red fox as an IH, along with the report of 100% vertical transmission in the latter (Almería et al., 2002; Zanet et al., 2023). These findings demonstrate that some populations can maintain the parasite cycle internally, without the intervention of other IH. Taken together, these findings support the dual role of wild canids in the *N. caninum* life cycle, with a more prominent contribution as intermediate hosts.

## 5 Conclusion

This review demonstrates that while *Canis latrans*, *C. lupus*, and *C. lupus dingo* are confirmed as DH of *N. caninum*, significant knowledge gaps persist concerning the natural dynamics in other wild canids. Methodological discrepancies between NIMs and EIMs, along with heterogeneity in sample preservation and analysis, engender challenges in interpreting results and comparing different studies.

In exposed canids, the evidence highlights the necessity of an integrated diagnostic approach (Serology, histopathology, IHC, and PCR) to fully understand their role in the parasite's life cycle.

Future research should prioritize: 1) Standardisation of diagnostic techniques and criteria 2) Expansion of geographic coverage to underrepresented regions with high wild canid diversity, particularly Africa, Asia and South America 3) Development of integrative study designs combining environmental sampling with molecular confirmation and phylogenetic analysis.

Furthermore, increasing both the geographical scale and the number of individuals evaluated is essential to strengthen the evidence on the role of wild canids in the biological cycle of *N. caninum*. Based on current knowledge, wild canids appear to play a more preponderant role as IH than as DH in the epidemiology of *N. caninum*.

## CRediT authorship contribution statement

**Agustina Soto-Cabrera:** Writing – review & editing, Writing – original draft, Investigation, Formal analysis, Data curation, Conceptualization. **Nathalia P. Scioscia:** Writing – review & editing, Visualization, Validation, Investigation, Conceptualization. **Dadin P. Moore:** Writing – review & editing, Visualization, Validation, Conceptualization. **Gastón Moré:** Writing – review & editing, Visualization, Validation, Supervision, Conceptualization.

## Conflicts of interest

We Agustina Soto-Cabrera, Nathalia P. Scioscia, Dadin P. Moore and Gastón Moré, authors from the manuscript intitled “**The role of wild canids in the epidemiology of *Neospora caninum*: a systematic review**” report no conflict of interest.
